# Above‐ and Belowground Traits are Linked Within Species, but Vary Differently Along Environmental Gradients

**DOI:** 10.1002/ece3.74109

**Published:** 2026-07-31

**Authors:** Robert Rauschkolb, Viktoria Dietrich, Barbara Knickmann, Birgit Nordt, Martin Freiberg, Isabell Hensen, Tim Meier, Ingeborg Lang, Jonathan Klaus Wenk, Lucienne Krause, Christine Römermann

**Affiliations:** ^1^ German Centre for Integrative Biodiversity Research (iDiv) Halle‐Jena‐Leipzig Leipzig Germany; ^2^ Institute of Biodiversity, Ecology and Evolution Friedrich Schiller University Jena Jena Germany; ^3^ Senckenberg Institute for Plant Form and Function Jena (SIP) Jena Germany; ^4^ Thünen‐Institute, Institute of Forest Ecosystems Eberswalde Germany; ^5^ Core Facility Botanical Garden University Vienna Vienna Austria; ^6^ Botanic Garden Berlin Freie Universität Berlin Berlin Germany; ^7^ Systematic Botany and Functional Biodiversity, Life Science Leipzig University Leipzig Germany; ^8^ Institute of Biology/Geobotany and Botanical Garden Martin Luther University Halle‐Wittenberg Halle (Saale) Germany; ^9^ Department of Functional and Evolutionary Ecology University of Vienna Vienna Austria

**Keywords:** climate change, field experiment, herbaceous species, root traits, SLA, trait‐based ecology

## Abstract

Plant functional traits are frequently used to compare species strategies and predict functions such as growth, reproduction, or phenology. They can also quantify intraspecific variation and thereby assess species' capacity to respond to environmental variation. While aboveground traits are well studied, comparatively little is known about belowground traits. In this study, we investigated the intraspecific response of above‐ and belowground plant traits to climatic and soil parameters by cultivating five perennial herbaceous species in experimental beds at five Central European botanical gardens. After up to four growing seasons we measured above‐ and belowground traits, and analysed their relationships with climatic conditions, soil chemistry and nutrient availability. We specifically tested whether above‐ and belowground trait pairs with similar functions show corresponding responses to environmental parameters. We found that above‐ and belowground traits are linked, with plant height negatively correlated with root branching and leaf dry matter content (LDMC) positively correlated with root dry matter content (RDMC). However, we found no evidence for a coupling between the leaf economic spectrum (LES) and the root economic spectrum (RES). Overall, intraspecific variation in response to environmental factors was mainly driven by nutrient availability, reflected in soil C/N ratios, whereas climatic differences played a less important role. Traits differed in the extent to which they were influenced by broad‐scale environmental conditions versus micro‐environmental variation. Our results demonstrate that even relatively small environmental differences can generate intraspecific variation in above‐ and belowground traits. This highlights the importance of measuring traits directly at study sites when applying trait‐based approaches. Although some easily measurable aboveground traits were linked to root traits in terms of function and environmental responses, we do not recommend using aboveground traits as proxies for root traits. The observed relationships were inconsistent, difficult to predict, and may depend on habitat conditions.

## Graphical abstract

Minor differences in environmental gradients or microhabitat variations can lead to intraspecific variation in above‐ and belowground traits. This highlights the importance of using traits measured on sites for trait‐based approaches. To some extent, easily measurable aboveground traits are linked to root traits in terms of their function and responses to environmental conditions, but we do not recommend using aboveground traits as a substitute for root traits in trait‐based approaches as the correlations are very difficult to predict and may depend on the habitat.

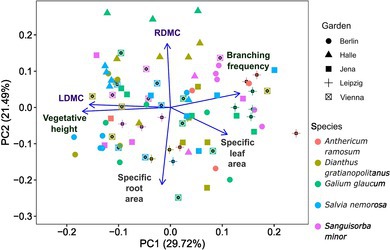

## 1 Introduction

Traits are of central importance in many areas of plant research (Westoby 2025). For example, they are used to compare ecological strategies among species, ecosystems and vegetation types (Choat et al. 2012; Díaz et al. 2016; Kunstler et al. 2016), as well as within species (Albert et al. 2010; Dwyer et al. 2014; Rosas et al. 2019). In addition, traits also play an important role in ecophysiology and functional ecology, as they are used to define species or individual‐specific plant growth, resource uptake and reproduction (Violle et al. 2007; Falster et al. 2018; Stears et al. 2022; Bucher et al. 2021; Sporbert et al. 2022).

Trait‐based ecology has largely focused on aboveground traits focusing on the costs of productive tissues, such as specific leaf area (SLA), leaf dry matter content (LDMC) and plant height (Kattge et al. 2020; Westoby 2025). Apart from aboveground traits, less is known about the role of root traits in trait‐based ecology (Weigelt et al. 2021), and studies combining above‐ and belowground plant traits are mostly done for tree species (e.g., Shen et al. 2019; Weemstra et al. 2020; Dietrich, Niederberger, Patriarca, et al. 2025), with few examples of herbaceous species (e.g., Liu et al. 2010; Mueller et al. 2024). When comparing aboveground and belowground traits, a frequently discussed question is whether there are key dimensions in both growth zones that reflect a trade‐off between resource acquisition and conservation, that is, whether there is both a leaf economic spectrum (LES) and a root economic spectrum (RES) (Reich et al. 1997; Wright et al. 2004; Shipley et al. 2006; Reich 2014). While some studies support the existence of a coordinated LES–RES framework, with analogous trade‐offs in leaves and roots (e.g., Reich et al. 1998; Freschet et al. 2010), others have reported weak or inconsistent support for a root economic spectrum, suggesting that root trait variation may not be captured by a single acquisitive–conservative axis (e.g., Comas and Eissenstat 2009; Kong et al. 2019). In principle, both economic spectra can be described by a wide range of traits (Wright et al. 2004; Freschet et al. 2010; Reich 2014). However, there are individual traits, such as specific leaf area (SLA), which already provide an adequate representation of the LES (Reich 2014; De la Riva et al. 2021). To compare aboveground and belowground resource‐acquisition strategies, homologous trait pairs are particularly informative because they capture analogous functions in leaves and roots (Reich et al. 1998; Freschet et al. 2010). Specific leaf area (SLA) and its belowground counterparts, specific root area (SRA) and specific root length (SRL), are especially suitable for such analyses, as all three traits are closely linked to resource acquisition efficiency and investment in acquisitive growth strategies (Pérez‐Harguindeguy et al. 2013; Freschet and Roumet 2017). In addition, leaf dry matter content (LDMC) and the homologous root dry matter content (RDMC) represent key traits describing the conservative end of the plant economic spectrum. Higher LDMC and RDMC values are generally linked to increased tissue longevity (leaves and roots, respectively), greater structural investment, and enhanced drought resistance (Pérez‐Harguindeguy et al. 2013; Laliberté 2016; Freschet and Roumet 2017; Rauschkolb et al. 2022; Reinelt et al. 2023). Besides the acquisitive–conservative spectrum, plant strategies can also be described along a size‐related axis that reflects competitive ability and resource foraging (Díaz et al. 2016). Along this axis, vegetative height and root branching density can be considered analogous above‐ and belowground traits. While plant height is associated with competition for light, root branching density reflects the capacity to explore and exploit soil resources. Together, these traits provide proxies for competitive resource acquisition above‐ and belowground (Keddy et al. 2002; Postma et al. 2014; Zhan and Lynch 2015; Jia et al. 2018).

Ongoing climate change is rapidly altering abiotic site conditions, which is for example indicated by more severe periods of reduced precipitation, such as the multi‐year drought from 2018 to 2020 in Central Europe (Rakovec et al. 2022), and consequently by more frequent reductions in soil nutrient availability (e.g., Sardans and Peñuelas 2005; Zuccarini et al. 2023). There are numerous studies that examine how environmental variability in terms of climate and soil properties drives intraspecific trait variation, with examples from forest systems (e.g., Braun et al. 2017; Dietrich et al. 2024; Dietrich, Niederberger, Frank, and Hauck 2025) and wild herbaceous species (Bucher et al. 2021; Deilmann et al. 2024). It can even be assumed that the changes in environmental parameters can lead to an increase in intraspecific differentiation of traits in the future (see Anderegg 2023; Westoby 2025).

This differentiation, in turn, can affect the aforementioned relationships between traits and plant functions. For example, the extent of the variation in SLA depends on water availability and temperature, whereas variation in LDMC is often only influenced by differences in temperature (Cochrane et al. 2016; Lang et al. 2019; Schuchardt et al. 2023 but see Pakeman 2013). Furthermore, differences in climate sensitivity, precipitation or mean annual temperature have been found to affect plant height (Moles et al. 2009, 2014; Lynn et al. 2023) and studies that included soil parameters identified soil pH and C/N ratio as a relevant predictor for SLA (e.g., Gong and Gao 2019). Although research has focused on the study of aboveground plant traits in response to changing environmental conditions, there are also examples of belowground traits. For example, root branching frequency (Postma et al. 2014; Zhan and Lynch 2015; Jia et al. 2018) and SRA (Hodge 2004, 2009; Freschet 2021; Dietrich, Niederberger, Patriarca, et al. 2025) were found to be related to changing nutrient and water availability in the soil. Depending on the other environmental circumstances, these changes have been reported to have either a negative or positive association with resource availability (Kermavnar et al. 2023; Dietrich, Niederberger, Patriarca, et al. 2025). Most studies analysed either above‐ or belowground traits, and comparative analyses are lacking (but see Ciccarelli et al. 2023). Nevertheless, certain links can be expected. For the pairs SLA vs. SRA as well as for vegetative height and root branching density, opposite reactions can be assumed in accordance with the optimal partitioning theory (Ågren and Franklin 2003; Kobe et al. 2010). Since belowground resources (e.g., water or nutrients) are less limited in more productive and competitive habitats than aboveground resources (e.g., photosynthetic active radiation, DeMalach et al. 2017), a positive relation for SLA and height and a negative for SRA and branching density can be expected. In contrast, for the pair LDMC and RDMC, it can be assumed that trait expression in response to harsh conditions such as drought is homologous (Stears et al. 2022; Sanaei et al. 2025).

With this study, we aimed to identify climatic and soil parameters that could have a particularly strong influence on intraspecific differentiation of plant traits, both above‐ and belowground. In particular, we investigated whether the above‐ and belowground trait pairs with equal functions (SLA vs. SRA, LDMC vs. RDMC, vegetative height vs. root branching density) show similar or different responses to varying environmental conditions. This work contributes to answering the question of the extent to which belowground traits should be considered individually in trait‐based approaches (Weigelt et al. 2021) or whether the use of easily measurable aboveground traits already provides sufficient information regarding plant strategies. For this purpose, we designed experimental fields with five perennial herbaceous species planted in beds at five Central European botanical gardens. This unique design allowed us to compare above‐ and belowground plant traits and their plasticity along climatic and soil gradients. Specifically, we tested the following hypotheses:

(1) Above‐ and belowground trait pairs with equal functions are strongly correlated, (2) with regard to the traits associated with acquisitive–conservative strategies, this indicates a link between LES and RES, (3) within species, trait expression shifts towards a more acquisitive strategy with increasing availability of belowground resources (i.e., soil nutrients and water), resulting in higher SLA, SRA, vegetative height, and root branching density. Conversely, decreasing soil water availability promotes a more conservative strategy, reflected by higher LDMC and RDMC. (4) Intraspecific trait variation is greater among gardens than within gardens, reflecting the larger environmental gradient among gardens.

## 2 Materials and Methods

### 2.1 Study Design

In 2019, we set‐up beds in four botanical gardens in Central Europe (Berlin (DE), Halle/Saale (DE), Jena (DE), and Vienna (AT), Table 1) with five subplots per garden (i.e., five replicates per garden). In 2020 we added another bed in Leipzig (DE). Each bed had a total area of 10–13 m^2^, with each of the five subplots measuring approximately 2 m^2^. The individual size depended on the circumstances in the respective botanical garden. All beds were filled with a locally prepared substrate mixture composed of limestone, sand and sandy‐loamy soil (50%–30%–20%). The substrate depth ranged from 25 to 30 cm, and all beds were exposed to full sun. This design created similar, although not identical, soil conditions among botanical gardens. In every subplot we planted five herbaceous perennial species of five plant families (*Anthericum ramosum*, *Dianthus gratianopolitanus* , *Galium glaucum* , *Salvia nemorosa* , and *Sanguisorba minor* ) in random mixtures, so that each species was planted in every of the five possible position across the five sub‐beds. The plants were grown in pots in the botanical gardens during spring using the local soil mixture of the beds and were planted in the beds as young plants at the beginning of summer (Age of the plants: approximately 5 months). Once a month we cleared the beds of wild weeds. Since the beds were exposed to natural climatic conditions, were generally calcareous and nutrient‐poor, and were only watered if necessary, we selected species that we assumed would generally cope well with nutrient deficiency and drought (i.e., dry grassland species). The amount of water given varied among gardens and was intended solely to prevent the plants from dying off during periods of intense summer heat. Planted individuals of each species originated from the same maternal populations or were even clones ( *A. ramosum* ), ensuring that the influence of genetic background on the measured traits was minimised.

**TABLE 1:** Summary of the climatic conditions and site parameters in the beds of the five botanical gardens.

	Berlin	Halle	Jena	Leipzig	Wien
Mean temperature^a^ (°C)	11.7	11.8	12.1	11.9	13.0
Precipitation sum^a^ (mm)	490	415	395	425	450
Elevation (m asl.)	45	92	157	120	180
Soil pH	8.68	7.88	8.53	8.39	8.53
Soil electronic conductivity (mS cm^−1^)	0.11	0.23	0.17	0.12	0.17
Soil N content (mg g^−1^)	0.05	0.20	0.12	0.09	0.09
Soil C/N	20.7	16.9	21.1	23.1	24.8

^a^ Mean temperature or precipitation sum between 20th Dec 2022 to 23rd Aug 2023. The temperature was measured using data loggers in the beds, and precipitation was extracted from data from nearby weather stations.

### 2.2 Climatic Data

In each botanical garden we installed loggers for recording temperature data at a height of 10 cm above the ground between 20th Dec 2022 and 23rd Aug 2023 (models used: TOMST TMS‐4, TOMST Thermologger & Hobbo Logger). We extracted the daily mean, maximum and minimum temperatures. We derived precipitation data for the gardens in Germany from the nearest weather station belonging to the Climate Data Center of the German Weather Service (www.cdc.dwd.de). For the Botanical Garden in Vienna, AT, we obtained data from the spartacus‐v2‐1d‐1 km dataset (www.data.hub.geospehere.at). Precipitation data was derived as the total daily amount for the same period in which the temperature loggers were active (20th Dec 2022 to 23rd Aug 2023, Table 1).

### 2.3 Aboveground Trait Measurements

During the 2023 growing season, at the time of peak flowering for each species, we measured vegetative height from the ground to the highest leaf and sampled healthy sun exposed leaves at mid plant height. These measurements were performed per individual per subplot; however, since the beds were established, some individuals had already died, so that the number of plants sampled was 107. In particular, many *Anthericum ramosum* plants have died; in Berlin, only three individuals could be sampled, none in Halle, and only one in Jena (see available data). After sampling, we measured the fresh weight of two leaves per individual and determined dry weight after 72 h of drying at 40°C. We calculated the LDMC by dividing leaf dry weight by its fresh weight. Remaining fresh leaves were scanned at a resolution of 300 dpi (CanoScan LiDE400, Canon Deutschland GmbH). To extract the leaf area, we used a python script (unpublished, Matthias Körschens) to identify the leaf pixels, which were then converted into cm^2^. We calculated the SLA by dividing the leaf area by the leaves' dry weight.

### 2.4 Belowground Trait Measurements

In autumn 2023, we excavated all surviving plants with a spade and a digging fork and froze the separated root systems until further processing. As the root measurements are very labor‐intensive and could only be analysed in a laboratory in Jena, we were unable to carry them out at the same time as the aboveground measurements. We carefully rinsed root systems under water, cut the fine roots according to the first three root order fractions (Freschet 2021), and measured their fresh weight. We scanned fine root fractions separately at a resolution of 1200 dpi using an Expression 12000XL (Pro) Scanner and the RhizoVision Software (Seethepalli et al. 2021) for the analysis of sample length, area and branching frequency (i.e., number of lateral roots formed per unit length of a parent root). Afterwards, we dried samples for at least 48 h at 70°C and determined the dry weight. We calculated SRA and RDMC by dividing root fraction surface area by its dry weight and root fraction dry weight by its fresh weight. We have chosen to use SRA rather than SRL for the analyses, as this trait better represents a potential RES (De la Riva et al. 2021).

### 2.5 Soil Sampling

During aboveground trait measurements, we took soil samples at the upper, middle and lower ends of the subplot at equal distances and at depths of 0 cm to 10 cm and 10 cm to 20 cm. We mixed the three samples of the same depth per subplot for further analysis. We dried mixed samples for 72 h at 40°C, sieved them twice (5 mm and 2 mm pores), and homogenised the sample with a mortar. Using a subsample of 5 mg mixed with deionised water, we measured pH and electronic conductivity (EC) (COMBI 5000, STEP Systems GmbH), and using 15 mg we measured C and N content (vario EL cube, Elementar Analysensysteme GmbH). For the statistical analyses described below, we calculated the means of both sampled soil depths. We use these three parameters as proxies for soil nutrient availability, as they provide information on how many nutrient ions are present (EC), how well nutrients are mobilised (pH) or directly how fertile the soil is (C:N ratio).

### 2.6 Statistical Analyses

We conducted all statistical analysis with the software R 4.4.2; means and corresponding standard error are presented throughout the paper. For non‐normal distributed data, which we checked using the Shapiro–Wilk test, we performed comparisons or correlations using either the Wilcoxon rank‐sum test or the Spearman correlation coefficient. To evaluate trait associations between above‐ and belowground traits across species, we conducted Spearman's correlations using the *stats*‐package (R Core Team 2024). Furthermore, we performed a principal component analysis (PCA) on z‐standardised trait values to investigate whether leaf economic spectrum (LES) and root economic spectrum (RES) traits are associated. To assess the effects of abiotic conditions on plant traits we used linear mixed‐effects models to assess the effects of abiotic conditions on plant traits using the R package *lme‐4* (Bates et al. 2015). For each plant trait, we analysed all species together and a maximum of 107 observations could be used for analysis. In all models, we included species identity and garden and subplot identity as random effects. As abiotic explanatory factors influencing the variability of the measured plant traits, we initially considered climatic parameters mean, maximum and minimum temperature as well as sum of precipitation and as proxies for belowground resources mean soil N and C content; the corresponding C/N ratio; and mean soil pH and electronic conductivity at subplot level. Afterwards we excluded minimum temperature, mean soil C and N content, and electronic conductivity from modelling due to strong correlation above 0.7 with other environmental factors. At the start of the modelling process, each model per plant trait included the full list of selected fixed effects that were not highly correlated. We used a backward stepwise selection for each model using the Akaike information criterion (AIC) for comparison (Dormann and Kühn 2009). Before and after the backwards stepwise selection, we checked residuals for normality using the R package *DHARMa* 0.4.6 (Hartig 2020), and we checked variance inflation to ensure it was below 4. If the residuals were not normally distributed, we transformed response variable by using the 2nd root order for the ratio of specific leaf area to root area and the 4th root order for root branching frequency. Although modelling showed that the influence of the random factors garden and subplot is very small, we kept the structure of the random effects the same for all models in order to account for the experimental design. To evaluate relative changes in trait pairs in response to the environment, we calculated the ratios of traits with comparable functions: the ratios SLA/SRA and LDMC/RDMC to assess the relationship between resource uptake efficiency and tissue longevity, and the ratio vegetative height/root branching to assess potential shifts in resource foraging. We then used these ratios to calculate the models described above in a corresponding way.

To identify the variability of traits within (i.e., across the five subplots) and among gardens, we used the ratio of interclass correlation coefficients (ICC) per level (within garden vs. among gardens). To do this, we first calculated linear mixed‐effects models per trait, including species identity as fixed effect and two random effects: (1) garden, to model variation among gardens; and (2) subplot nested within garden, to model variation within gardens. The according variation is expressed as variance proportions in percentage. Afterwards, we calculated the ICC‐ratios (Equation 1), whereas a ratio between 0 and 1 implies a higher variation among gardens and a ratio between −1 and 0 a higher variation within a garden.

(1) $$\mathrm{ICC}\;\text{ratio}=\frac{\%\text{expl}.\;\text{Variance among Gardens}-\%\text{expl}.\;\text{Variance within Gardens}}{\%\text{expl}.\;\text{Variance among Gardens}+\%\text{expl}.\;\text{Variance within Gardens}}.$$

To estimate the variance of the ICC ratios, we used 500‐fold bootstrapping and drew 5 samples per site for each repetition.

## 3 Results

### 3.1 Associations Between Aboveground and Belowground Plant Traits

The five species examined were in general very similar in terms of their trait composition and differed only slightly in SLA and LDMC (Figure S1). The Spearman's correlations between above‐ and belowground traits revealed different significant trait associations (Table 2). A higher SLA occurred together with higher RDMC and branching frequency. LDMC was positively correlated with RDMC and vegetative height negatively correlated with branching frequency. When comparing the correlations between above‐ and belowground plant traits with the correlations within above‐ or belowground plant traits, we found only higher correlations for vegetative height with SLA (ρ = −0.28, *p* < 0.05; Table S1) or LDMC (ρ = 0.43, *p* < 0.05; Table S1), and they were of comparable strength within the belowground parameters (Table S1).

**TABLE 2:** Spearman's correlation coefficient^a^ among aboveground and belowground plant traits of *Anthericum ramosum*, *Dianthus gratianopolitanus* , *Galium glaucum* , *Salvia nemorosa* , and *Sanguisorba minor* (*N* = 91 observations).

	Specific leaf area	Leaf dry matter content	Vegetative height
Specific root area	0.19 (*)	0.15	0.05
Root dry matter content	0.26*	0.23*	−0.008
Branching frequency	0.25*	−0.12	−0.24*

^a^ Levels of significance: (*) *p* ≤ 0.10, *p ≤ 0.05.

**FIGURE 1:**
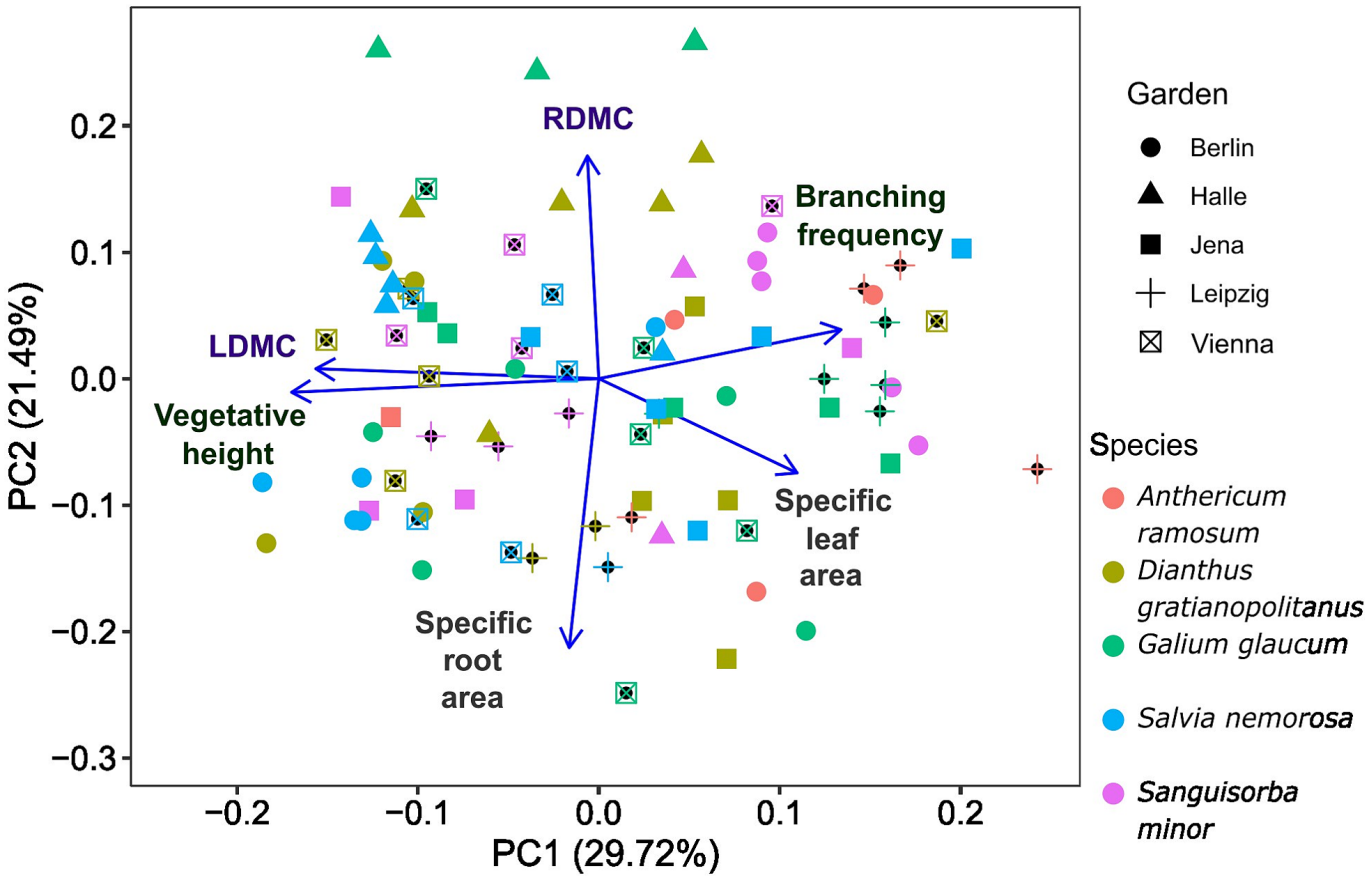
Principal component analysis among plant traits of *Anthericum ramosum*, *Dianthus gratianopolitanus* , *Galium glaucum* , *Salvia nemorosa* , and *Sanguisorba minor* at all Botanical gardens (*N* = 91 observations).

For the PCA with z‐standardised trait values we relied on data of 91 individuals sharing measurement of all six investigated traits. The principal component analysis made clear that PC1 is described in particular by the trade‐off between SLA and LDMC, and PC2 by that between SRA and RDMC (Figure 1).

### 3.2 Influences of Abiotic Conditions on Plant Traits

**FIGURE 2:**
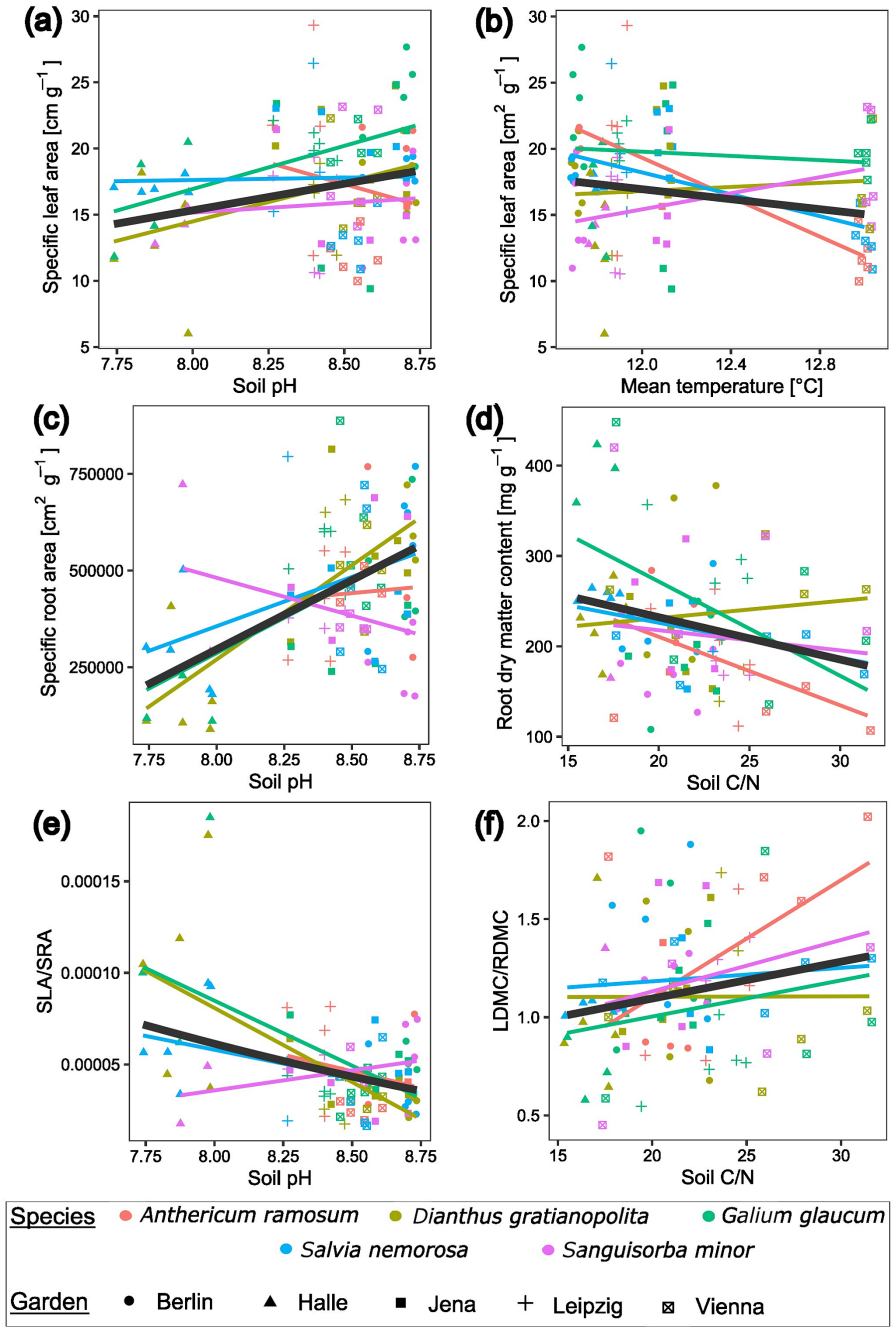
Significant associations between specific leaf area, soil pH (a), and mean temperature (b), between soil pH and specific root area (c), soil C/N ratio and root dry matter content (d), soil pH and the ratio between specific leaf and root area (e), and the ratio between leaf and root dry matter content (f). The black regression lines represent the mean effect observed across species. Results correspond to linear mixed‐effects models shown in Tables S2 and S3 (*N*_per species_ = 20–25).

Linear mixed‐effects models for each plant trait and the corresponding ratios revealed different significant patterns in response to abiotic conditions. In general, the impact of botanical garden identity or subplot identity was lower than that of species identity (Figure 2; Tables S2 and S3). Of all traits analysed, those relating to the description of specific surface areas were found to be the most dependent on abiotic conditions. In contrast, vegetative height and root branching frequency showed the least dependency (Tables S2 and S3). SLA and SRA increased significantly with higher soil pH (Figure 2a,c; Table S2a). Lower mean temperature was associated with a significantly higher SLA, whereas a higher maximum temperature tended to increase the SRA (Figure 2b,c; Table S2a). Meanwhile, the ratio between SLA and SRA decreased with an increase in soil pH, but was unaffected by any temperature‐related variables (Figure 2e; Table S2a).

With increasing soil pH, the RDMC decreased and the ratio between LDMC and RDMC increased significantly (Figure 2d,f; Table S2b). However, the LDMC was not dependent on the soil C/N ratio (Table S2b). We found no links between intraspecific trait variability and precipitation.

### 3.3 Comparison of Trait Variability Within and Among Gardens

**FIGURE 3:**
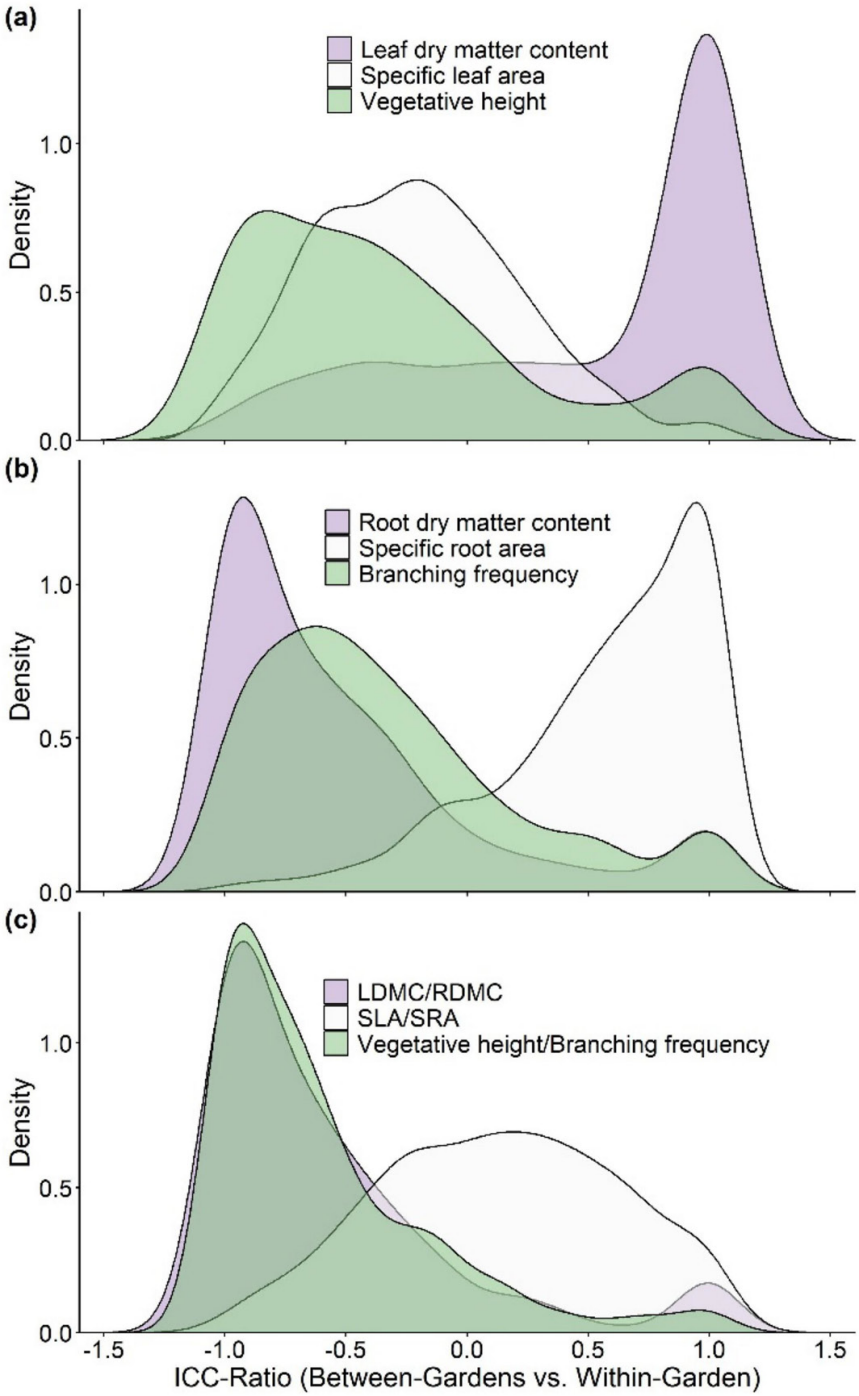
Density of interclass correlation coefficients based on 500‐fold bootstrapping showing the variation among vs. within gardens for aboveground (a) and belowground (b) plant traits and their ratios (c). ICC‐ratios between 0 and 1 indicate a higher variation among gardens and a ratio between −1 and 0 indicate a higher variation within a garden.

The trait variability among and within beds for the trait pair indicating resource foraging (vegetative height and root branching frequency) was consistent (Figure 3a,b). For both traits, the variation within a garden was always higher than among gardens. This also applied to the ratio of both traits (Figure 3c). In contrast, differences were found between the traits in relation to the ICC‐ratio for the pairs SLA vs. SRA and LDMC vs. RDMC. While SRA, LDMC, and LDMC/RDMC showed higher variations among gardens, SLA, RDMC, and SLA/SRA were more variable within a garden (Figure 3a–c).

### 3.4 Abiotic Conditions in the Five Botanical Gardens

Among gardens, differences in abiotic conditions are mostly driven by soil pH, N content, and electronic conductivity, as well as maximum temperature (Table 1; Figure S2). Soil C/N, mean temperature, and precipitation sum explained less differences. In detail, the soil pH was lowest in Halle and highest in Berlin. The other three gardens showed more similar values, although there were significant differences between Leipzig and Vienna (*p* < 0.05; Figure S3a). Electronic conductivity showed the opposite pattern, with comparable levels in Jena and Vienna, while Leipzig had similarly low values to Berlin (*p* < 0.05; Figure S3b). On average, Halle had the highest soil N content, followed by comparable levels in Jena, Vienna, and Leipzig, and significantly lower levels in Berlin (*p* < 0.05; Figure S3c). The C/N ratio was lowest in Halle, but comparable in all other gardens (*p* < 0.05; Figure S3d).

## 4 Discussion

By cultivating the same five herbaceous species in experimental plant beds across five Central European botanical gardens, we showed that above‐ and belowground traits were generally correlated. However, we found no evidence for a coupling between the leaf economic spectrum (LES) and the root economic spectrum (RES). In this study, resource foraging was characterised by two traits, vegetative height and root branching frequency, which were negatively correlated, while adaptation to harsh conditions such as drought was characterised by LDMC and RDMC, which were positively correlated. Intraspecific variation in traits in response to different environmental conditions was generally lower than interspecific variation. Overall, soil nutrient availability had a stronger impact on trait variations than differences in temperature and precipitation. We also found that some traits are more variable due to greater environmental differences among botanical gardens, and others are more variable due to micro‐environmental differences within a garden.

### 4.1 Above‐ To Belowground Trait Associations

The expected correlations between homologous below‐ and aboveground traits were partially confirmed. LDMC and RDMC, as well as vegetative height and branching density, were strongly correlated. SLA and SRA were also correlated, but this relationship was only marginally significant. In terms of tissue longevity and drought tolerance (LDMC vs. RDMC), we observed no trade‐off between above‐ and belowground organs, as we found a positive correlation. This observation is consistent with our hypothesis that adaptations to harsh conditions, such as drought, occur in a homologous manner in both growth zones (Stears et al. 2022; Sanaei et al. 2025). In contrast, we found a trade‐off for the trait pair describing resource foraging, as vegetative height and branching density were negatively correlated. This can be explained by the fact that the resources available for growth must be divided between root growth and aboveground growth. For example, large vegetative height is beneficial when a plant grows in aboveground competition for light, whereas a branched root system should be beneficial for nutrient‐poor or heterogeneously distributed nutrient habitats (Keddy et al. 2002; Postma et al. 2014; Zhan and Lynch 2015; Jia et al. 2018).

It was unexpected that SLA and SRA correlate positively, albeit only slightly. In the context of optimal partitioning theory (Ågren and Franklin 2003; Kobe et al. 2010), we have expected a negative correlation. When evaluating the results, it must be considered that the correlations between these two traits can depend heavily on site conditions. For example, Liu et al. (2010) found that SLA and SRL (another proxy for belowground resource uptake efficiency) were positively correlated in 64 herbaceous species growing in the semi‐arid and arid regions of northern China. Our study sites were all exposed to full sun, received little additional watering and were generally nutrient‐poor (Figure S3). It is therefore reasonable that the relationship between SLA and SRA does not correspond to the optimal partitioning theory, as the study system reflected a semi‐arid habitat (Liu et al. 2010).

We found that SLA can be considered a key trait for herbaceous plants when analysing above‐ and belowground traits as it is also correlated with RDMC and branching density in addition to SRA. A higher RDMC reduces mortality risk during drought (Konôpka et al. 2007; Stears et al. 2022), and a higher branching density of roots helps to reach water in deeper soil layers. These adaptations are particularly important for plants that grow fast (i.e., having a high SLA), to be supplied with sufficient water and nutrients (Reich 2014; De la Riva et al. 2021). It is interesting to note that the frequently observed negative correlation between SLA and LDMC (Pérez‐Harguindeguy et al. 2013; Sporbert et al. 2022) is not reflected in the relationship between SLA and RDMC, despite the positive correlation between LDMC and RDMC. This finding may also help explain why we found no evidence for a relationship between the leaf economic spectrum (LES) and the root economic spectrum (RES) (Figure 1), consistent with previous studies (e.g., Comas and Eissenstat 2009; Kong et al. 2019). To summarise, we can state that the correlations between above‐ and belowground traits do not generally display the expected pattern. Nevertheless, SLA appears to be an important leaf trait that is linked to various root traits.

### 4.2 Trait‐Environment Associations

Overall, our results showed that the impact of environmental conditions on the intraspecific variability of the measured traits is limited. Of the 45 potential trait‐environment relationships, only six were statistically significant (Figure 2). This finding is consistent with previous studies suggesting that traits are often only weakly associated with environmental variation (Anderegg 2023; Westoby 2025). As environmental gradients covered in our study were relatively narrow (Table 1, Figure 2), it remains unclear whether stronger patterns would emerge across a broader range of environmental conditions. However, evidence from studies encompassing larger gradients showed that intraspecific trait responses may remain limited. For example, Dwyer et al. (2014) found that, despite a pronounced increase in precipitation across a spatio‐temporal gradient in Australia, SLA responded significantly in only 25 of 51 herbaceous species. Likewise, although gradients in soil nitrogen availability and pH were considerably larger than those observed in our study, significant intraspecific variation in SLA was detected in only a small subset of species. In contrast, controlled greenhouse experiments frequently reveal trait variation even in response to relatively small environmental differences (Poorter et al. 2009), highlighting that environmental effects on trait expression tend to be less pronounced under natural field conditions (Poorter et al. 2016; Anderegg 2023; Karitter et al. 2026).

Nevertheless, we found significant patterns of variation in trait expressions based on relatively small environmental differences, such as an annual average temperature of about 1°C. It can be assumed that the negative correlation between SLA and temperature found in our study is not solely attributed to this single environmental factor, but rather to a combination of factors, as higher temperatures, for example, expose plants to greater drought stress (Feng and Fu 2013; Rauschkolb et al. 2024), which leads to a reduction in SLA (Dwyer et al. 2014; Cochrane et al. 2016; Gong and Gao 2019). However, the five study sites do not differ greatly in terms of precipitation (Table 1). Since we applied only a minimum of watering, it can be assumed that any potential increase in drought is not due to large climatic patterns or maintenance, but rather to local, small‐scale conditions. This assumption is also confirmed by the ICC ratios (Figure 2a).

Slight differences in soil depth, soil texture, and plant‐induced shading among subplots can affect local water availability. As a result, variation in water availability at the microsite scale may explain a larger proportion of the observed trait variation than the combined effects of precipitation and temperature at the study site. In our study, soil parameters related to nutrient availability were more important for trait expression than temperature or precipitation (see Ordoñez et al. 2009; Gong and Gao 2019), as we found five associations between soil parameters and trait variation, but only one significant relationship between a plant trait and temperature (Figure 2). With increasing nutrient availability, for example, characterised by low C:N ratios or neutral soil pH, an increase in growth and resource uptake can be expected (Ordoñez et al. 2009). We found that SLA and SRA positively correlated with increasing pH (Figure 2a,c), which contrasts with a meta‐study by Gong and Gao (2019) that found SLA was lower under increasing pH in 3700 species. However, since all species studied are associated with alkaline soils (Tichý et al. 2023), we assume that the slight higher values in pH, leads to more optimal environmental conditions for the studied species, which positively correlates with both SLA and SRA (Kermavnar et al. 2023). This highlights how species‐specific the relationships between variability in abiotic factors and the resulting variability in traits can be. Another finding is that SRA is more strongly influenced by differences in soil pH than SLA (Figure 2e). Since the variation in pH is particularly influenced by differences among the botanical gardens, this may explain why the ICC ratio for SRA is > 1 (i.e., variability among gardens is greater than within gardens). This is reasonable, as SRA can exhibit significant variability due to soil conditions (Hodge 2004, 2009; Freschet 2021). Therefore, SRA may have captured variability both among and within gardens.

Regarding the trait pair describing tissue longevity (LDMC vs. RDMC), we found that with increasing availability of nitrogen, fine roots become less durable overall (Figure 2d), also in relation to the leaves (Figure 2f), indicating that more biomass is invested in tissue density and stability of leaves than in roots. This observation is consistent with other studies indicating that plants with sufficient nutrient supply invest in fast‐growing and less long‐lived fine roots (Fischer et al. 2019). While LDMC showed higher variations among gardens, RDMC was more variable within a garden (Figure 2a,b), we conclude that LDMC is overall more strongly influenced by larger scale differences (i.e., temperature influencing drought potentially linked to watering or rain before harvest) and RDMC is more strongly influenced by microhabitat influences (i.e., uneven distribution of nutrients in the soil) or differences in competition patterns (Bassi et al. 2025).

For the two traits describing resource foraging (vegetative height vs. root branching), we found no significant correlations between the measured environmental parameters and trait variability (Table S3). This result is consistent with greater variability within gardens than among gardens (Figure 2). We conclude that these two traits are particularly influenced by biotic factors such as competition (Freschet 2021; First et al. 2025), which varied across the subplots due to differences in the cover and size of the co‐growing species (personal observations).

While the study design enabled the detection of intraspecific associations between environmental conditions and trait expression among gardens and within gardens, it does not permit causal inference. As environmental factors were not experimentally manipulated, the observed patterns should be interpreted as correlations. Future experimental studies that manipulate individual environmental variables will be required to determine causal relationships. An additional consideration is that the four‐year duration of the experiment may have allowed feedbacks between plants and their environment, particularly the soil, to develop over time. The extended study period enabled perennial plants and their overwintering organs to become fully established, thereby better reflecting natural conditions than short‐term or pot experiments. This in turn may also have increased plot heterogeneity through plant‐driven environmental changes and cumulative management effects, reducing experimental control.

## 5 Conclusion

Despite the relatively small environmental gradient between the five botanical gardens, we observed significant intraspecific variation in some aboveground and belowground traits. This highlights how important it is for trait‐based studies not to rely on averages or values from databases (e.g., TRY; Kattge et al. 2020), but to carry out site‐specific measurements. Several aboveground and root traits correlated with one another, and some showed similar relationships with environmental conditions. However, these relationships were generally weak, varied depending on the trait and species, and we were unable to find evidence of coordinated strategies regarding resource acquisition and conservation. Our results therefore suggest that aboveground traits provide only limited information about the variation in belowground traits within the grassland species and environmental context studied. We suggest that future experimental and field studies covering broader environmental gradients, habitats and species pools are required to improve our understanding of the relationships between aboveground and belowground traits, as well as their associations with environmental conditions.

## Author Contributions

**Robert Rauschkolb:** data curation (supporting), formal analysis (lead), investigation (supporting), methodology (lead), supervision (lead), visualization (lead), writing – original draft (lead), writing – review and editing (lead). **Viktoria Dietrich:** formal analysis (lead), visualization (lead), writing – original draft (lead), writing – review and editing (lead). **Barbara Knickmann:** conceptualization (lead), investigation (supporting), methodology (lead), resources (supporting), supervision (supporting), writing – review and editing (supporting). **Birgit Nordt:** conceptualization (lead), investigation (supporting), methodology (lead), resources (supporting), supervision (supporting), writing – review and editing (supporting). **Martin Freiberg:** conceptualization (lead), investigation (supporting), methodology (lead), resources (supporting), supervision (supporting), writing – review and editing (supporting). **Isabell Hensen:** conceptualization (lead), investigation (supporting), methodology (lead), resources (supporting), supervision (supporting), writing – review and editing (supporting). **Tim Meier:** data curation (lead), investigation (lead), writing – review and editing (supporting). **Ingeborg Lang:** investigation (supporting), resources (supporting), supervision (supporting), writing – review and editing (supporting). **Jonathan Klaus Wenk:** data curation (lead), investigation (lead), visualization (supporting), writing – review and editing (supporting). **Lucienne Krause:** data curation (lead), investigation (lead), visualization (supporting), writing – review and editing (supporting). **Christine Römermann:** conceptualization (lead), investigation (supporting), methodology (lead), resources (supporting), supervision (supporting), writing – review and editing (supporting).

## Funding

This work was supported by Deutsche Forschungsgemeinschaft (Grant 118) and Deutsches Zentrum für integrative Biodiversitätsforschung Halle‐Jena‐Leipzig (Grants 09159715 and 09159723).

## Conflicts of Interest

The authors declare no conflicts of interest.

## Supporting information


**Table S1:** Spearman's correlation coefficient^a^ between aboveground (a) and belowground (b) plant traits of *Anthericum ramosum*, *Dianthus gratianopolitanus* , *Galium glaucum* , *Salvia nemorosa* , and *Sanguisorba minor* (*N* = 107 observations).
**Table S2:** Results of linear mixed‐effects models (model estimates ± SE)^a^ analysing the effect of soil chemistry, temperature and precipitation^b^ on (a) specific leaf area, specific root area, and their ratio and (b) leaf dry matter content, root dry matter content, and their ratio of *Anthericum ramosum*, *Dianthus gratianopolitanus* , *Galium glaucum* , *Salvia nemorosa* , and *Sanguisorba minor* (*N*_leaf_ = 107, *N*_roots_ = 96, *N*_ratio_ = 95).
**Table S3:** Results of linear mixed‐effects models (model estimates ± SE)^a^ analysing the effect of soil chemistry, temperature and precipitation^b^ on vegetative height (*N* = 104), root branching frequency (*N* = 96), and their ratio (*N* = 92) of *Anthericum ramosum*, *Dianthus gratianopolitanus* , *Galium glaucum* , *Salvia nemorosa* , and *Sanguisorba minor* .
**Figure S1:** Comparison of (a) aboveground traits, (b) belowground traits, and (c) ratios between aboveground and belowground traits between selected species (*N*_per species_ = 25). Different letters indicate significant differences, *p* ≤ 0.05, Wilcoxon rank‐sum test.
**Figure S2:** Principal component analysis among abiotic characteristics of subplots in botanical gardens (*N* = 25).
**Figure S3:** Distribution of soil characteristics shown as the mean across different soil depth (0–10 cm; 10–20 cm) per subplot for each botanical garden (*N* = 25): pH (a), electronic conductivity (b), N content (c) and C/N ratio (d).

## Data Availability

The data used for the analyses are published under the following https://doi.org/10.5281/zenodo.17864595.
