# Supplementary material for: Above‐ and Belowground Traits are Linked Within Species, but Vary Differently Along Environmental Gradients
Source: Ecol Evol. 2026 Jul 31;16(8):e74109. doi: 10.1002/ece3.74109 (PMC13428029; doi:10.1002/ece3.74109)
Supplement: Supplementary file 1 — Table S1: Spearman's correlation coefficienta between aboveground (a) and belowground (b) plant traits of Anthericum ramosum, Dianthus gratianopolitanus , Galium glaucum , Salvia nemorosa , and Sanguisorba minor (N = 107 observations). Table S2: Results of linear mixed‐effects models (model estimates ± SE)a analysing the effect of soil chemistry, temperature and precipitationb on (a) specific leaf area, specific root area, and their ratio and (b) leaf dry matter content, root dry matter content, and their ratio of Anthericum ramosum, Dianthus gratianopolitanus , Galium glaucum , Salvia nemorosa , and Sanguisorba minor (N leaf = 107, N roots = 96, N ratio = 95). Table S3: Results of linear mixed‐effects models (model estimates ± SE)a analysing the effect of soil chemistry, temperature and precipitationb on vegetative height (N = 104), root branching frequency (N = 96), and their ratio (N = 92) of Anthericum ramosum, Dianthus gratianopolitanus , Galium glaucum , Salvia nemorosa , and Sanguisorba minor . Figure S1: Comparison of (a) aboveground traits, (b) belowground traits, and (c) ratios between aboveground and belowground traits between selected species (N per species = 25). Different letters indicate significant differences, p ≤ 0.05, Wilcoxon rank‐sum test. Figure S2: Principal component analysis among abiotic characteristics of subplots in botanical gardens (N = 25). Figure S3: Distribution of soil characteristics shown as the mean across different soil depth (0–10 cm; 10–20 cm) per subplot for each botanical garden (N = 25): pH (a), electronic conductivity (b), N content (c) and C/N ratio (d). [file ECE3-16-e74109-s001.docx]

**Supplementary Material to**

**Above- and belowground traits are linked within species, but vary differently along environmental gradients**

Robert Rauschkolb^1,2,3, *,†^ & Viktoria Dietrich^4,†^ & Barbara Knickmann^5^, Birgit Nordt^6^, Martin Freiberg^1,7^, Isabell Hensen^1,8^, Tim Meier^8^, Ingeborg Lang^9^, Jonathan Klaus Wenk^2^, Lucienne Krause^2^, Christine Römermann^1,2,3^

**Table S1:** Spearman`s correlation coefficient^a^ between aboveground (a) and belowground (b) plant traits of *Anthericum ramosum*, *Dianthus gratianopolitanus*, *Galium glaucum*, *Salvia nemorosa*, and *Sanguisorba minor* (*N*=107 observations).

| 1. **Aboveground traits** | | |
| --- | --- | --- |
|  | Leaf dry matter content | Vegetative height |
| Specific leaf area | -0.19 (*) | -0.28 ** |
| Leaf dry matter content | - | 0.43 *** |
| 1. **Belowground traits** | | |
|  | Root dry matter content | Branching frequency |
| Specific root area | -0.24 * | 0.07 |
| Root dry matter content | - | 0.23 * |

^a^ Levels of significance: (*) *P*≤0.10, * *P*≤0.05, ***P*≤0.01, ****P*≤0.001.

**Table S2:** Results of linear mixed-effects models (model estimates ± SE)^a^ analyzing the effect of soil chemistry, temperature and precipitation^b^ on (a) specific leaf area, specific root area, and their ratio and (b) leaf dry matter content, root dry matter content, and their ratio of *Anthericum ramosum*, *Dianthus gratianopolitanus*, *Galium glaucum*, *Salvia nemorosa*, and *Sanguisorba minor* (*N*_leaf_=107, *N*_roots_=96, *N*_ratio_=95).

| **(a)** | **Specific area** |  |  |
| --- | --- | --- | --- |
|  | Leaf | Root | Leaf / root^c^ |
| Intercept | 17.42 ± 0.70 *** | 0.00004 ± 0.0002 *** | 0.0070 ± 0.0003 *** |
| Soil pH | 1.18 ± 0.46 * | 0.00001 ± 0.0002 *** | -0.0007 ± 0.0002 * |
| Soil C/N | - | - | - |
| Mean temperature | -0.89 ± 0.41 * | - | - |
| Maximum temperature | - | 0.0004 ± 0.0002 (*) | - |
| Precipitation sum | - | - | - |
| Conditional R^2^ | 20% | 20% | 22% |
| Marginal R^2^ | 8% | 19% | 15% |
| Random effects *σ^2^* | | | |
| Garden | 0.02 × 10^-6^ | 0 | 0.0002 × 10^-3^ |
| Garden:Subplot | 0.66 | 0 | 0 |
| Species | 1.52 | 0.0004 × 10^-4^ | 0 |
| Residual | 0.16 | 0.0003 × 10^-6^ | 0.0002 × 10^-2^ |
| **(b)** | **Dry matter content** |  |  |
|  | Leaf | Root | Leaf / root |
| Intercept | 237.66 ± 6.91 *** | 0.230 ± 0.010 *** | 1.13 ± 0.04 *** |
| Soil pH | -2.51 ± 6.06 | - | - |
| Soil C/N | - | -0.020 ± 0.007 * | 0.07 ± 0.03 * |
| Mean temperature | - | - | - |
| Maximum temperature | -8.48 ± 5.94 | - | -0.06 ± 0.04 (*) |
| Precipitation sum | - | - | - |
| Conditional R^2^ | 13% | 13% | 8% |
| Marginal R^2^ | 3% | 6% | 7% |
| Random effects *σ^2^* | | | |
| Garden | 38.97 | 0.005 × 10^-2^ | 0 |
| Garden:Subplot | 0 | 0.005 × 10^-10^ | 0 |
| Species | 127.73 | 0.003 × 10^-1^ | 0.001 |
| Residual | 1461.60 | 0.004 | 0.110 |

^a^ Levels of significance: (*) *P*≤0.10, * *P*≤0.05, ***P*≤0.01, ****P*≤0.001.

^b^ Daily mean (temperature) or daily sum (precipitation) between the 20th Dec 2022 to the 23rd Aug 2023.

^c^ The ratio of specific leaf area to specific root area was transformed using the 2^th^ root to remove a left-skewed distribution and to account for normally distributed residuals.

**Table S3:** Results of linear mixed-effects models (model estimates ± SE)^a^ analyzing the effect of soil chemistry, temperature and precipitation^b^ on vegetative height (*N*=104), root branching frequency (*N*=96), and their ratio (*N*=92) of *Anthericum ramosum*, *Dianthus gratianopolitanus*, *Galium glaucum*, *Salvia nemorosa*, and *Sanguisorba minor*.

|  | Vegetative Height | Branching frequency^c^ | Vegetative height/ Branching frequency |
| --- | --- | --- | --- |
| Intercept | 40.18 ± 3.04 *** | 0.440 ± 0.004 *** | 111.60 ± 10.92 *** |
| Soil pH | - | 0.005 ± 0.003 | - |
| Soil C/N | - | - | - |
| Mean temperature | - | -0.004 ± 0.003 | - |
| Maximum temperature | - | - | - |
| Precipitation sum | 4.38 ± 2.23 (*) | - | 12.06 ± 6.53 (*) |
| Conditional R^2^ | 7% | 10% | 11% |
| Marginal R^2^ | 3% | 4% | 3% |
| Random effects *σ^2^* | | |  |
| Garden | 0 | 0.005 × 10^-2^ | 0 |
| Garden:Subplot | 0 | 0 | 20.44 |
| Species | 19.78 | 0.002 × 10^-2^ | 347.71 |
| Residual | 525.91 | 0.007 × 10^-1^ | 4219.55 |

^a^ Levels of significance: (*) *P*≤0.10, * *P*≤0.05, ***P*≤0.01, ****P*≤0.001.

^b^ Daily mean (temperature) or daily sum (precipitation) between the 20th Dec 2022 to the 23rd Aug 2023.

^c^ Branching frequency was transformed using the 4^th^ root to remove a left-skewed distribution and to account for normally distributed residuals.


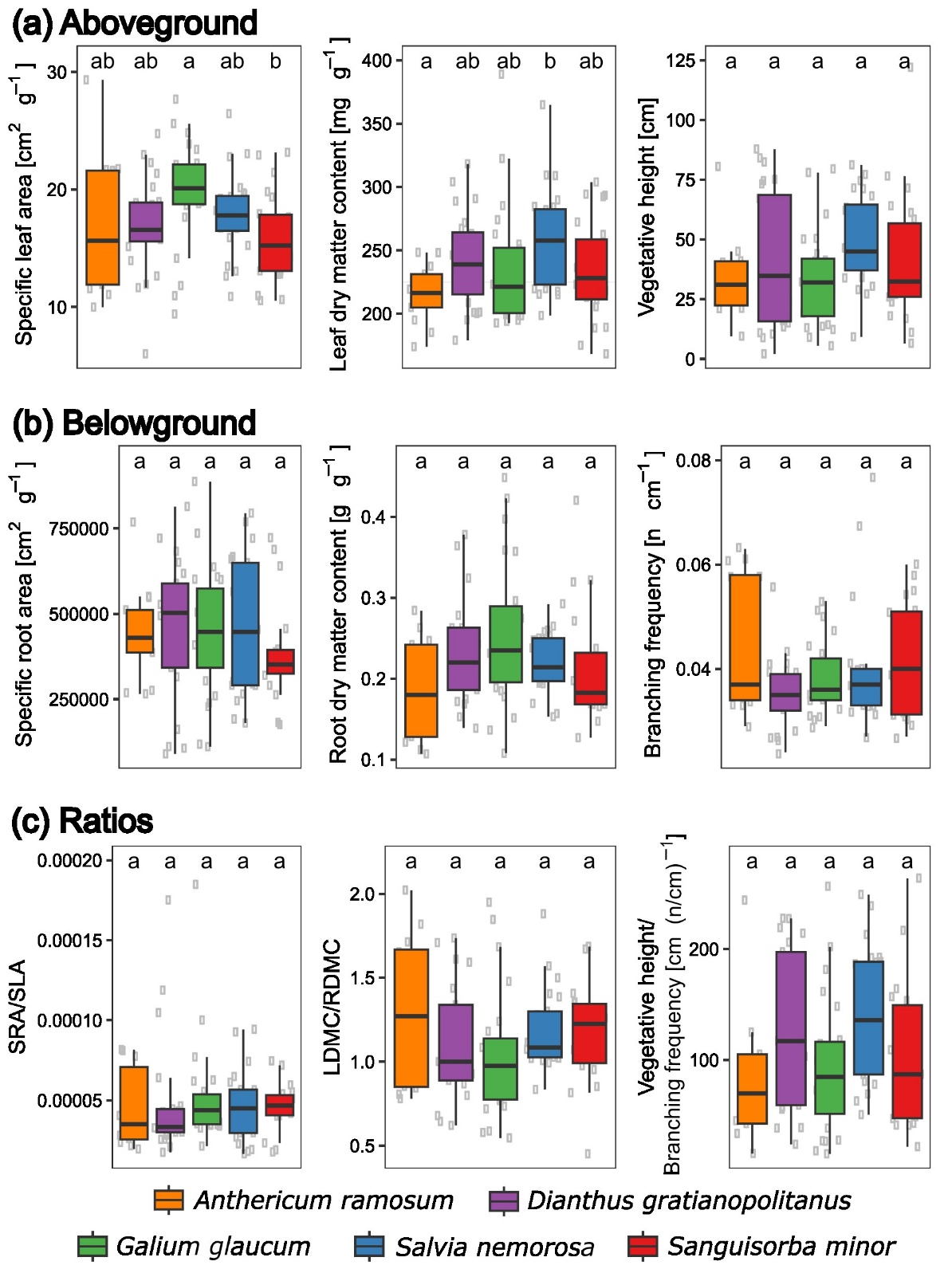


**Figure S1**: Comparison of (a) aboveground traits, (b) belowground traits, and (c) ratios between aboveground and belowground traits between selected species (*N*_per species_= 25). Different letters indicate significant differences, *P*≤0.05, Wilcoxon rank-sum test.


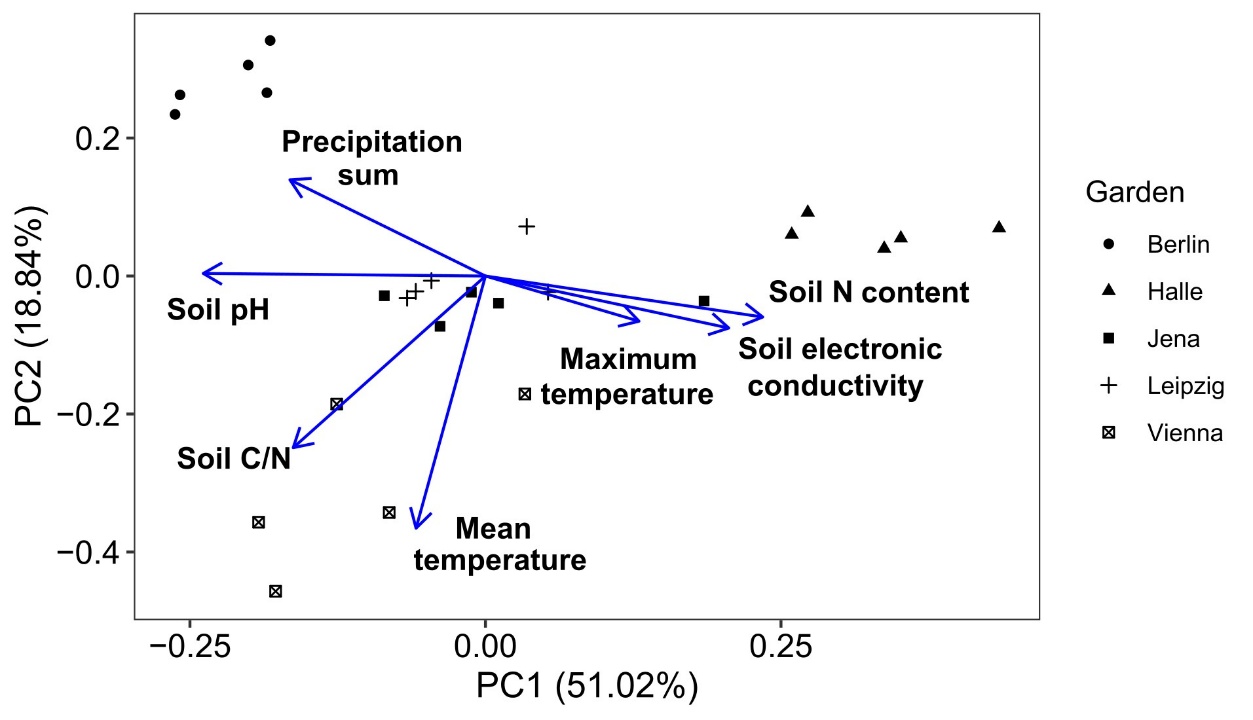


**Figure S2**: Principal component analysis among abiotic characteristics of subplots in botanical gardens (*N*=25).


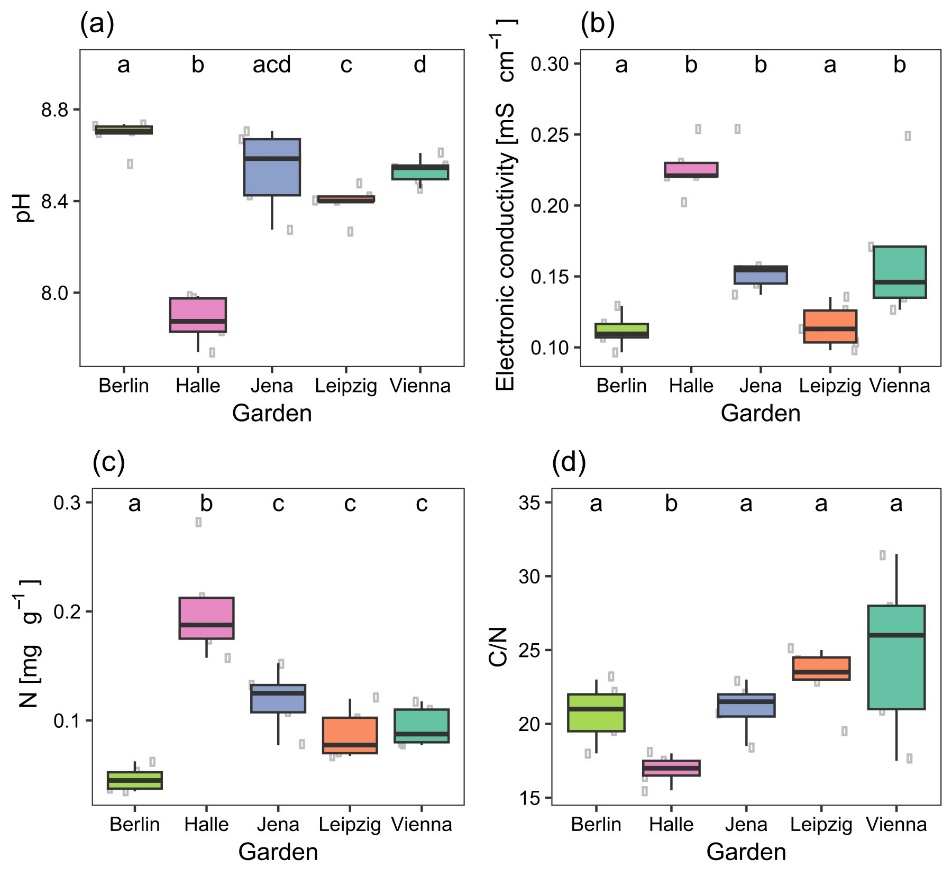


**Figure S3**: Distribution of soil characteristics shown as the mean across different soil depth (0 – 10 cm; 10 – 20 cm) per subplot for each botanical garden (*N*=25): pH (a), electronic conductivity (b), N content (c) and C/N ratio (d).
